# Unexpected Event Prediction in Wire Electrical Discharge Machining Using Deep Learning Techniques

**DOI:** 10.3390/ma11071100

**Published:** 2018-06-28

**Authors:** Jose A. Sanchez, Aintzane Conde, Ander Arriandiaga, Jun Wang, Soraya Plaza

**Affiliations:** 1 Aeronautics Advanced Manufacturing Center, CFAA (UPV/EHU), Bizkaia Technology Park, Building 202, 48170 Zamudio, Spain; 2 Machine-Tool Institute (IMH), Azkue Auzoa 1 48, 20870 Elgoibar, Spain; aintzane@imh.eus; 3 iCub Facility, Istituto Italiano di Tecnologia Via Morego, 30, 16163 Genova, Italy; ander.arriandiaga@gmail.com; 4 Faculty of Mechanical Engineering, Tianjin University of Science & Technology (TUST), Dongjiang Rd, Hexi Qu, Tianjin 300222, China; jwang003@ikasle.ehu.eus; 5 Faculty of Engineering of Bilbao, UPV/EHU, Plaza Torres Quevedo 1, 48013 Bilbao, Spain; soraya.plaza@ehu.eus

**Keywords:** WEDM, deep learning, deep neural networks, Industry 4.0

## Abstract

Theoretical models of manufacturing processes provide a valuable insight into physical phenomena but their application to practical industrial situations is sometimes difficult. In the context of Industry 4.0, artificial intelligence techniques can provide efficient solutions to actual manufacturing problems when big data are available. Within the field of artificial intelligence, the use of deep learning is growing exponentially in solving many problems related to information and communication technologies (ICTs) but it still remains scarce or even rare in the field of manufacturing. In this work, deep learning is used to efficiently predict unexpected events in wire electrical discharge machining (WEDM), an advanced machining process largely used for aerospace components. The occurrence of an unexpected event, namely the change of thickness of the machined part, can be effectively predicted by recognizing hidden patterns from process signals. Based on WEDM experiments, different deep learning architectures were tested. By using a combination of a convolutional layer with gated recurrent units, thickness variation in the machined component could be predicted in 97.4% of cases, at least 2 mm in advance, which is extremely fast, acting before the process has degraded. New possibilities of deep learning for high-performance machine tools must be examined in the near future.

## 1. Introduction

Machine tools and in general, manufacturing industries, have traditionally been characterized by relying on empirical approaches when it comes to process optimization. Due to the large number of phenomena and variables involved in each operation, the practical application of theoretical models is difficult. In fact, although theoretical models are very interesting for understanding the underlying physical phenomena, they usually exhibit important limitations for industrial practice.

This fact is particularly evident in the case of manufacturing components for high-added value sectors, such as aircraft manufacturing. The aerospace industry has experienced an exponential increase in recent years. It is expected that by 2032 there will be double the total number of aircraft worldwide [1]. This trend has generated great investment by manufacturing companies in order to adapt their products to the increasing tolerance and accuracy requirements that are demanded by this sector. Unconventional machining processes have gained acceptance and within them, much attention has been directed towards wire electrical discharge machining (WEDM). This technology allows for the processing of difficult-to-cut, extremely hard materials with very tight tolerances and with impressive surface finish [2,3]. Nonetheless, trial and error approaches are still required for process optimization due to the above-mentioned limited accuracy of theoretical models [4]. In this context, artificial intelligence (AI) and more specifically, deep learning (DL) techniques appear to be an interesting approach, provided that massive amounts of data can be collected from the process.

Deep learning using Deep Neural Networks (DNNs) has achieved impressive state-of-the-art results in very difficult learning tasks such as image recognition [5], handwriting recognition [6], natural language processing [7], image description [8], and mitosis detection [9]. In contrast to shallow neural networks (SNNs), in a DNN, a series of hidden layers extract abstract features from a sequence or images [10]. Because of this, an overwhelming number of new applications are being developed in the field of information and communication technologies (ICTs), including automatic translation and voice recognition, thus increasing the interest from both academia and industry. A brief summary of the main network architectures for deep learning is presented in the following paragraphs.

For processing sequential data, recurrent neural networks (RNNs) are a common approach in many fields [10]. The earliest attempts to train RNNs were made by Rumelhart et al. using back-propagation through time [11]. Later, Elman introduced the Elman network with feedback from the output of the hidden layer to the input of said layer [12]. However, these training methods and architectures do not deal properly with long-term time dependencies due to vanishing and exploding gradients [13]. Thus, in order to solve the vanishing gradients problem, in 1997 Hochreiter & Schmidhuber introduced the long short-term memory networks (LSTMs) [14]. Unlike the classic RNN, an LSTM uses gates to decide whether or not to keep the existing memory [15]. Thus, an LSTM unit is able to keep an important feature over a long distance and therefore, deal with long-term time dependencies. Although variations of the LSTM architecture have been proposed, probably the most commonly used is the gated recurrent unit (GRU) that replaces the input, forget, and output gates by an update gate and a reset gate, reducing the number of gates from 3 to 2 [16].

To extract features from sequences of data, convolutional neural networks (CNNs) exhibit outstanding performance. CNNs are feed-forward neural networks that combine three ideas: local receptive fields, subsampling, and shared weights [17]. The local receptive fields and subsampling ideas were already in the neocognitron neural network model proposed by Fukushima [18]. By using CNNs with local receptive fields, neurons can extract features from images (2D structures), sequences, or time series (1D structures). From a convolutional layer, multiple futures can be extracted using several future maps. Furthermore, by combining these features in the subsequent layers, CNNs are capable of detecting higher-order features. Generally, each convolutional layer is followed by a subsampling layer that reduces the resolution of the feature map to reduce the sensitivity of the output to distortions [19]. Unlike the neocognitron, a CNN is trained with the back-propagation technique. Thus, weight sharing reduces the number of free parameters, improving generalization.

When looking at industrial applications outside the cope of ICTs, DNNs have been traditionally used in fault diagnosis for various sectors. For instance, Yin et al. [20] presented a novel method for fault diagnosis in high-speed railways, which is currently based on manual operation. In particular, the authors proposed an automated diagnosis network in order to detect failures in vehicle-on-board equipment. The results show that a deep belief network outperforms other trained networks and improves the accuracy of fault diagnosis by up to 95%. A further illustration can be found in the selection of different techniques for improving fault diagnosis in rolling bearings [21]. Following a thorough study of different AI techniques, the authors concluded that rule based method could become an extremely versatile tool in the fault diagnosis of rotating machinery.

Efficient training of deep neural networks is only possible if a massive number of labeled data is available to apply back-propagation training algorithms and this is not always possible in manufacturing environments. Though, some interesting approaches can be found in scientific literature. Most published research is devoted to optimization of process parameters in advanced machining techniques (such as laser cutting, electro-chemical machining, ion beam micro-milling, and grinding) using SNNs [22,23,24,25]. Only a very limited number of studies have examined the use of DL in machining. In a very interesting recent study, Wang developed a DL based approach to material removal rate prediction in polishing technologies [26]. A pattern recognition, identification, and process control system has also been developed by Gunter to achieve an intelligent laser-welding machine [27].

To the knowledge of the authors, none of the published research focusing on the WEDM process has addressed process modeling using deep learning techniques. Selection of optimum process parameters has been a classic application of SNNs [28], which have also been used [29] to extract information about degradation of the cutting conditions during WEDM. In a more recent study, Conde et al. [30] proposed using a variant of SNNs to predict the accuracy of components machined by WEDM. By combining the predictions of the network with the simulated annealing optimization technique, wire paths of variable radii can be designed so that radial deviations due to wire deformations can be minimized. The results revealed that the average deviation between network predictions and actual components is below 6µm, which falls within the current limits of process accuracy. The search and recognition of behavioral patterns of voltage and current signals in the WEDM process has been studied by Caggiano et al. [31,32], who presented a SNN that effectively correlates voltage and current signals with the defects and marks originated on the machined component during the WEDM process. In all cases, the success of the network exceeded 81%.

At this point, it must be highlighted that during the WEDM process, extremely large amounts of data can be collected using high-frequency voltage and current probes. Data from every single discharge during the process can be collected and patterns that contain useful information about the actual machining process, no matter process conditions, can be examined. Taking into account the efficiency shown by DNNs in tasks related to pattern recognition, in this work an original contribution to advance unexpected event prediction during practical WEDM operations using deep learning techniques is presented. The occurrence of an unexpected event, namely a change of the thickness of the machined part, can be predicted in advance by recognizing hidden patterns from process signals. Raw data are directly obtained from the machining process carried out in industrial conditions by using voltage and current probes and a high-frequency oscilloscope. Various DNN architectures have been studied and it was found that the combination of a convolutional layer with gated recurrent units achieved the best performance. By adopting this approach, thickness variation can be predicted in 97.4% of cases, at least 2 mm in advance, which is fast enough as to act before the WEDM process is degraded. New possibilities for applying DNNs in the field of advanced manufacturing and high-performance machine tools must therefore be examined in the future.

## 2. Materials and Methods

### 2.1. Instrumentation and Measured Variables

The WEDM erosion mechanism is based on the generation of discrete short discharges between two electrically conductive electrodes (wire and workpiece) in a fluid dielectric medium. In a recent paper, Almacinha et al. [33] proposed the feasible possibility that in the sinking electrical discharge machining (EDM) process, with hydrocarbon oil as a dielectric, multiple discharges occur during pulses with long on-time. However, in the case of ire WEDM, the short duration of the pulses (1.2 μs in commercial machines as the one used in the experiments) and the use of deionized water as a typical dielectric medium, the possibility of occurrence of multiple discharges during one pulse has not yet been proven. Therefore, in this work, the common hypothesis in WEDM [2] that workpiece material removal mechanism is attributed to the consecutive occurrence of discharges was used. As each single discharge generated a crater of a few micrometers on the workpiece, the combined contribution of millions of these resulted in the removal of part material, thus drawing the shape of the part [34].

**Figure 1:**
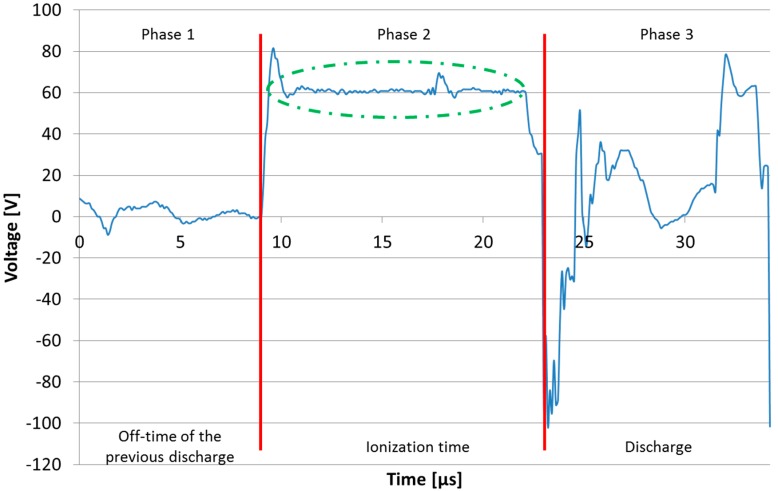
Voltage signal evolution during a single discharge in an industrial wire electrical discharged machining (WEDM) operation.

Figure 1 shows voltage evolution during a single discharge, as collected in an actual WEDM operation. A discharge can be divided into three parts. Before a discharge occurred (Phase 1, Figure 1), an off-time (voltage zero) was programmed, during which the gap between electrodes was cooled down and dielectric flow tried to remove the debris resulting from the previous discharge. In Phase 2 (Figure 1) the isolating capacity of the dielectric (deionized water in this case) was locally broken by the application of a voltage (commonly known as open-circuit voltage, see Table 1) between the wire and workpiece. The open-circuit voltage was applied by the machine generator. Then, ionization started (voltage signal was constant and current was zero amperes). This period, known as ionization time, was not controlled by the machine generator but by the local conditions of the dielectric. In other words, ionization time was not a machine parameter: for each single discharge, it depended on the specific electrical conductivity conditions of the dielectric. If flushing was effective and the gap was clean, ionization time was long. On the contrary, if flushing was difficult and debris was present in the gap, ionization time was short or even zero. Finally Phase 3 (Figure 1), when the electrical local conductivity of the dielectric between wire and workpiece was high enough (ionization ended), voltage dropped and current flowed during the on-time, resulting in part material removal due to the generated heat. For the experiments carried out, discharge current during on-time was 5A (see Table 1). This general pattern was reproduced during the process, although it is difficult to model phenomena such as the presence of debris between the electrodes, since differences were introduced between the discharges, affecting process performance.

Each single discharge contained valuable information about process performance. As shown in the previous section, other authors have recently attempted to correlate process signals with final part quality. However, advanced pattern recognition may be far more efficient in evaluating process performance. The fact that large amounts of information can be collected during the process (with sampling rates as high as 10.0 MS/s, as explained below) opens the possibility of training DNNs that have already proven their excellence in other fields (see Section 1).

**Figure 2:**
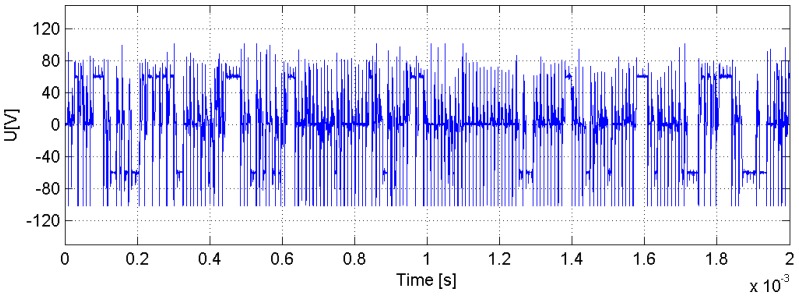
Example of voltage data sequence.

Voltage sequences were acquired during WEDM using a high-frequency oscilloscope Tektronix DPO5034B (Tektronix UK Ltd., Berkshire, UK) and a voltage probe Tektronix TMDP0200 (Tektronix UK Ltd., Berkshire, UK) connected to the WEDM machine. Figure 2 shows an example of voltage signal measurement. The sampling rate was 10.0MS/s, with a resolution of 100 ns. This is required to record any possible event during ionization of the discharge channel. A significant number of consecutive discharges must be recorded, because, as shown in Figure 2, there was a certain degree of variability between them. Therefore, a signal length of 200 µs was chosen. Measuring range for the voltage probe was set at −120 V to +120 V. The reason for this was that, although open-circuit voltage was set at 60 V (see Table 1), some random voltage peaks appeared and they would also be recorded. Also, commercial WEDM machines implement the feature of ensuring zero average voltage to avoid parasite currents that may affect process performance. This is why wire polarity changed as shown in Figure 2.

### 2.2. Experimental Methodology: WEDM Tests

In order to simulate the conditions of a degraded WEDM operation, a typical industrial situation in which process parameters cannot be controlled in advance was reproduced in our experiments. During an industrial operation, WEDM process parameters are set by machine-table look-up. Those parameters apply to a given combination of factors including part material, part thickness, and machining time. The parameters are available in the machine, and have been obtained through controlled experiments by the machine manufacturer. The machine user therefore finds in the WEDM machine the optimum combination of parameters for his/her application.

However, during WEDM cutting, operation conditions may vary. A typical example is an unexpected variation in part thickness. Parameters can be changed on-line once thickness change has happened [35] but anticipation of thickness change before it occurs has not been addressed. In fact, this feature is not present in commercial machines.

**Figure 3:**
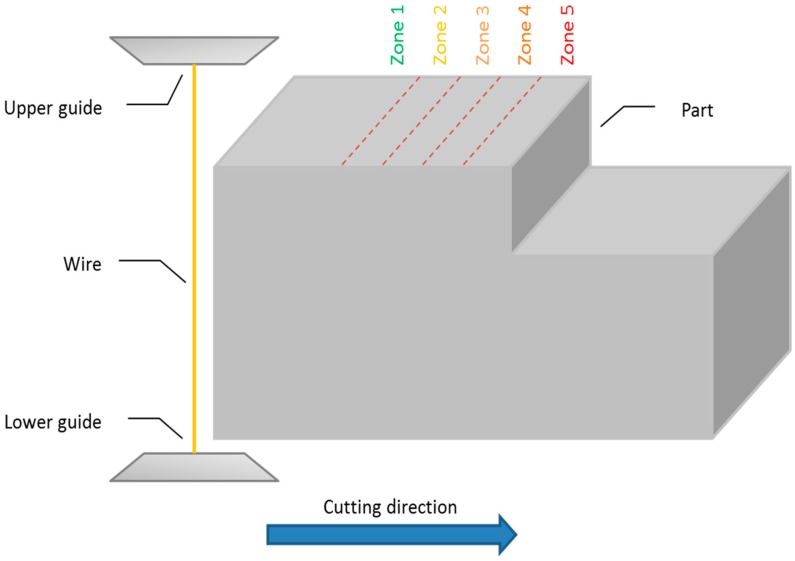
Scheme of the process with the sample geometry.

Controlled experiments were designed and carried out involving variation of part thickness during a WEDM operation. Part material for the samples was AISI D2 (ISO 160CrMoV12) 62 HRc tool steel, quenched and tempered. Stepped sample parts were prepared for the experiments, in which the WEDM cut faced a sudden thickness variation from 100 mm to 80 mm. Figure 3 shows a scheme of the process during the cutting of the test part.

The experiments were conducted under industrial conditions using commercial WEDM machinery (ONA AX3 WEDM machine, ONA Electroerosión S.A., Durango, Spain). The wire used was an uncoated brass wire (CuZn37) of 0.25 mm diameter, with ultimate strength of 900 N/mm^2^ and 1% elongation. As explained previously, WEDM electrical parameters (listed in Table 1) were selected by machine table look-up, and they correspond to roughing conditions.

When the wire approached the point of thickness change, the cut began to degrade because dielectric pressure was lost. This resulted in the occurrence of different voltage patterns in the discharges with respect to those occurring when the cut was performed under optimum conditions. To the best of our knowledge, there is not yet an industrial system that can detect such a pattern change. In order to conduct a systematic analysis, voltage sequences were collected at different distances from the point of thickness change. To do so, five different zones were defined: 5 mm away (Zone 1) from the point of thickness change, 4 mm away (Zone 2), 3 mm away (Zone 3), 2 mm away (Zone 4), and 1 mm away (Zone 5). The closer the wire to Zone 5 (in other words, to the point of thickness change), the more degraded the cut will be. 

Hence, five different zones of 1 mm length were established in order to adequately describe the cutting process degradation. The recording length was set to 0.8 mm, so that the oscilloscope could be reset during the remaining time. This allowed for a total of 567 sequences of 2 ms to be recorded, with a resolution of 100 ns and a sample rate of 10.0 MS/s. Thus, mean values of 140 discharges per sequence were recorded. Furthermore, this process was repeated 16 times in order to accumulate an appropriate number of tests.

These collected data were used to generate three different datasets. The first was used to study different DNN architectures to classify the voltage sequences of each zone. The second dataset was used to check the performance of the DNN to classify the sequences for Zones 1, 3, and 5. Finally, the third dataset was used to check the performance of the DNN for the slightly less ambitious task of classifying the sequences of the first and fifth zones. The Z_all dataset was balanced, so that there are an equal number of sequences for each class. For the other two cases, all of the available data were used in order to avoid significantly reducing the training dataset. However, the number of samples for each class was similar and there was little difference between the zones, as can be seen in Table 2.

### 2.3. Deep Learning Architectures Tested

The efficiency of different DNN architectures was evaluated by dividing the Z_all dataset into training (70%), validation (15%), and testing (15%) categories. The training, validation, and testing datasets were balanced, i.e., there was an equal number of sequences from each zone. All models were trained up to 100 epochs with categorical cross-entropy loss function and Adam optimizer [36]. Once the model was trained, the performance of the model was measured using the testing dataset, evaluating precision, recall, and F1 score.

The DNN models evaluated were a CNN, an RNN, and a bidirectional RNN and CNN combined with RNN. For the RNN, the GRU was used since it has recently been shown [34] that GRU slightly outperformed vanilla LSTM on almost most tasks and because the GRU is faster to train. The model studied is the following:

- CNN: the first layer was a convolutional layer with 50 filters of 10 × 1 dimension (10 × 1 × 50). The signals were of one dimension and, therefore, a 1D convolutional layer was used. The following layer was a stacked convolutional layer composed of 100 layers of 10 × 1 dimensions. After these two layers, a max pooling layer was used to down sample the input by two. Moreover, a dropout of 0.2 was also used to avoid overfitting. A further two convolutional layers of 150 filters with a smaller dimension (5 × 1) were then used. In this case a max-pooling layer was also used to down sample by two and dropout. The fully connected layer of 150 neurons with ReLu and dropout was used. Finally, a softmax activation function was used in the last layer. In all convolutional layers, a ReLu is used.
- GRU: in the case of the model with GRUs, the model had three layers of 50, 50, and 25 units with dropout of 0.2 intercalated between the layers to avoid overfitting. Finally, the last layer was fully connected with a softmax activation function.
- Bidirectional GRU: the model with bidirectional GRU layers was quite similar to the GRU model but with fewer units in each layer. Thus, the model was composed of three bidirectional GRU (BiGru) of 10, 50, and 25, with 0.2 dropout intercalated and softmax activation function in the last fully connected layer, as in the GRU model.
- Convolutional layer + GRU: the last model had a convolutional first layer to extract features from the signals, followed by two layers of GRU units. Hence, the first convolutional layer had 100 filters of 10 × 1 dimension followed by a max pooling of 2 for down sampling with a dropout and ReLu. Two GRU layers of 150 and 50 with dropout were then stacked. Finally, as in the case of both the GRU and BiGRU models, a fully connected layer with softmax activation function was used.

## 3. Results and Discussion

The results of Table 3 clearly show that the architecture combining a convolutional layer and GRU network outperformed other models for all the metrics analyzed. Therefore, for the other datasets (z_135 and z_15) this model was used to analyze the performance of the model and the complexity of the dataset. Due to the reduced dataset for classifying between Zones 1 and 5 and the fact that selecting the best model was not among the aims of the current study, the dataset was divided into training (70%) and testing (30%).

The results achieved with fewer zones (Table 4) were much higher than those yielded from all the zones. Moreover, the F1 Score for Z_135 dataset was 0.9169 and for Z_15 was 1. These results were outstanding, as they highlight the ability of the CGRU (Convolutional Gated Recurrent Unit) network to classify voltage sequences with high accuracy. The models with GRU units clearly outperformed those with CNN. This appeared logical because current research has indicated that GRU units deal accurately with sequences. In fact, CNN without any gate unit cannot satisfactorily classify WEDM spark sequences with a F1 score lower than 60%. Therefore, for classifying WEDM spark sequences, it is highly recommended to use DNNs with GRU units.

Focusing on models with GRU units, it is interesting to see that, in terms of precision, the model with bidirectional GRU achieved almost the same result as the GRU model but outperformed the GRU in terms of recall and F1 Score. This is interesting because the BiGRU model has less GRU units in the input layer (10 in the BiGRU and 50 in the GRU). However, the results were not sufficiently clear to draw the conclusion that for sequence classification, a BiGRU model would outperform the GRU model in all cases.

Similarly, analyzing the results from Table 4, it appears that adding a convolutional layer in the input of a GRU model helped to classify WEDM spark sequences. Thus, the first convolutional layer helped to extract features from spark sequences and then GRU units modeled these new sequences generated by the convolutional layer. Therefore, the results show that a CGRU model works accurately when classifying WEDM sequences with high precision (0.7260). Moreover, Table 4 shows that this model classified almost perfectly when dealing with less complicated datasets. Indeed, the model was capable of achieving 100% precision for classifying sequences of Zones 1 and 5.

**Figure 4:**
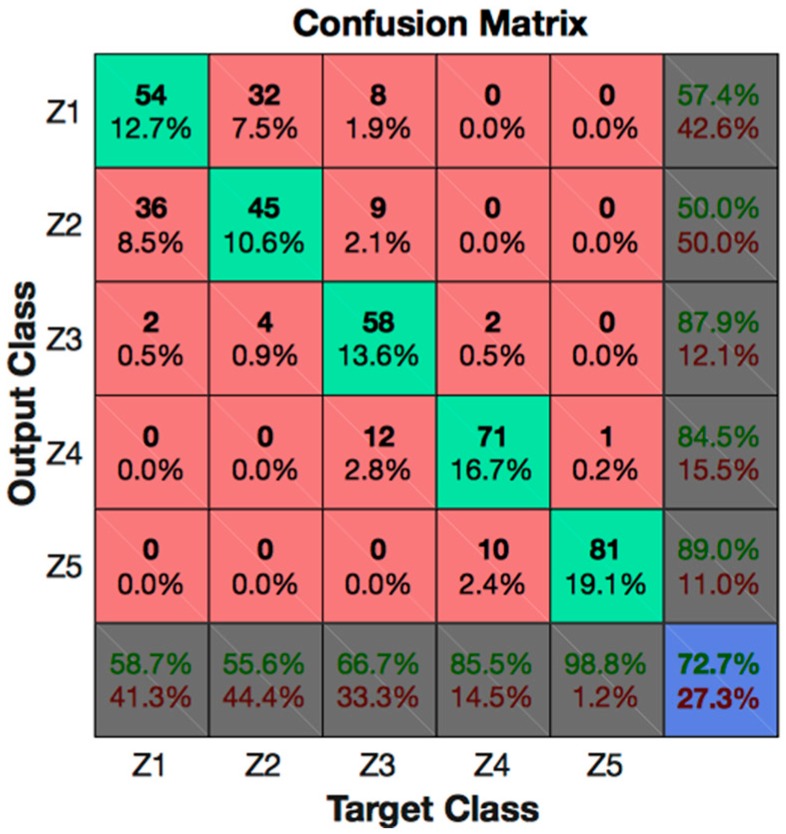
Confusion matrix for Z_all datasets with the CGRU model.

In contrast, from the process point of view, these results can be analyzed as follows. As the wire got closer to the thickness variation point, the behavior of the signal varied. This can be observed in the confusion matrixes of Figure 4. As explained in Section 2.2, Zone 1 (Z1) was the one that described a stable process, while Zone 5 (Z5) was the one closest to the point at which the thickness change occurred.

Figure 4 displays the confusion matrix considering the available data for the five zones (Z_all dataset). It can be observed that there was no interference between Zones 1 and 2 and Zones 4 and 5 or vice versa, or between Zones 3 and 5. From this result it can be stated that, when the cutting process came to be unstable, this was always successfully detected in advance by the neural network. For the experiment carried out, since the average feed for part thickness 100 mm was 1.4 mm/min, this means that there were at least 1.4 min to act before thickness variation occurred. Clearly, this time decreased when part thickness was smaller because of the higher feed, but in any case it would be tens of seconds (about 30 s when part thickness is 50 mm), which is more than enough time to make corrective actions. Moreover, the misclassification between zones was less than 3% among all consecutive zones throughout the degradation process, excluding Zones 1 and 2 (in any case, less than 10%), which was the starting point of the cutting degradation process.

**Figure 5:**
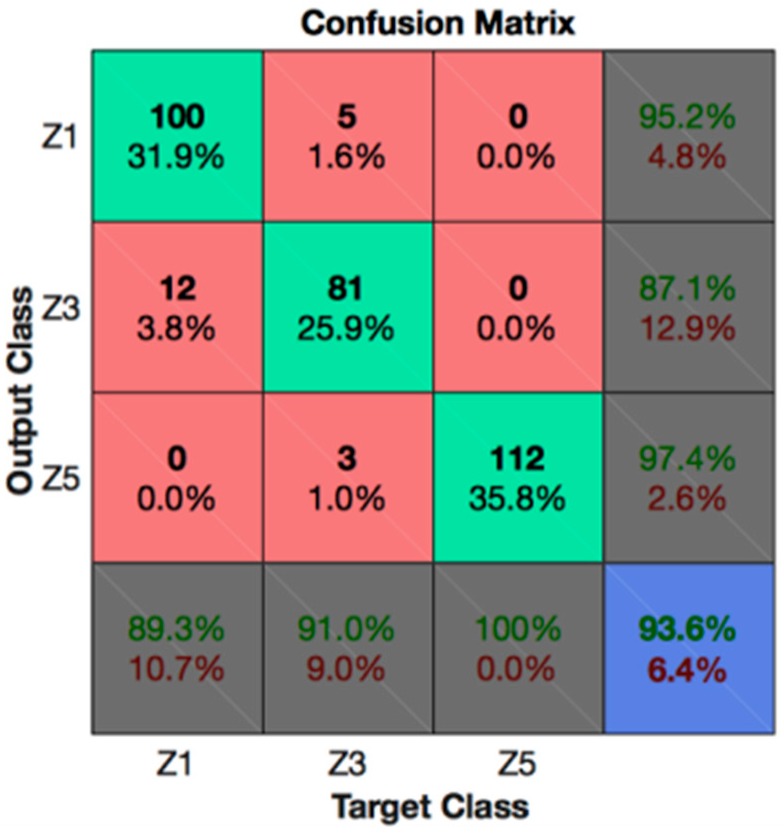
Confusion Matrix for Z_135 dataset with CGRU model.

Figure 5 shows the confusion matrix results when only Zones 1, 3, and 5 were used, that is, an stable cutting zone, an intermediate degraded zone and finally, the one in which the thickness variation occurred. Again, the first and most obvious consideration was that there is no confusion between Zones 1 and 5, which means that a stable cut was clearly distinguished from the nearest region to the thickness variation point. The mix between Zones 1 and 3 occurred in less than 4% of the cases; however, it is more likely that a degraded cut is confused for a stable cut than vice versa. Finally, it is worth noting that there were 1% of cases in which Zone 5 was classified as Zone 3. In conclusion, even if in 4% of the cases a thickness variation of 4 mm could not be predicted in advance, this change can always be predicted 2 mm earlier than it occurs.

To conclude, to the extent that the regions have been arbitrarily chosen, a variation in the behavioral pattern between a stable cut and a degraded cut was clearly shown in both cases (Z_all dataset and Z_135 dataset). Thus, the DNN enabled rapid action to be taken during the WEDM process.

Finally, Goodfellow et al. [10] stated that in supervised deep learning, to achieve acceptable results, more than 5000 labelled examples per category are needed and with at least 10 million labelled examples it is possible to match or exceed human performance. However, in this study, only 567 labelled examples for each category were available, considerably less than the recommended 5000. Although from a process point of view the results are excellent, one might consider that there is much room for improvement, because with more labelled examples, deeper ANNs can be used to reach higher precision results. However, as stated in the introduction, due to the difficulties in collecting large amounts of data in machine-tool workshops, in many cases it is not feasible to use DNNs. Therefore, if improvements in deep learning are to be exploited to their full potential for use in pattern recognition, then the companies and researchers involved in manufacturing and data collection must play a role in ensuring that new strategies are put in place.

## 4. Conclusions

The aim of this study was to evaluate the possibility of predicting an unexpected event during an industrial WEDM machining process by using DNNs to recognize hidden patterns from process raw voltage signals. A precision, recall, and F1 score comparison for different DNN models and datasets was provided. The results clearly showed that a model with a first convolutional layer with two GRU layers outperformed the other models. Moreover, this model achieved outstanding performance for the other datasets, with a precision of around 100%. From the process point of view, confusion matrixes showed that thickness variation can be predicted, at least 2 mm in advance, which allows sufficient time to act on machining parameters. New possibilities for applying DNNs in the field of advanced manufacturing and high-performance machine tools must be examined in the future. In particular, given the difficulty in collecting large quantities of labeled examples from machining processes, new strategies will need to be developed to resolve this problem. When large amounts of labeled data become available, the possibility of extensively applying DNNs in manufacturing will become a reality.

## Figures and Tables

**Table 1:** Electrical parameters as selected by machine table look-up.

WEDM Parameters	Settings
Height [mm]	100
Off-time [µs]	9.0
On-time [µs]	1.2
Current intensity [A]	5.0
Open-circuit voltage [V]	60.0
Initial dielectric pressure [bar]	17.0
Wire tension [kg]	1.2

**Table 2:** Dataset used.

Denotation	Zones	Sequences
Z_all	1, 2, 3, 4, 5	2835 (5 × 567)
Z_135	1, 3, 5	2088 (688 + 677 + 723)
Z_15	1, 5	1411 (688 + 723)

**Table 3:** Model results for Z_all dataset.

Model	Precision	Recall	F1 Score
CNN	0.5806	0.5765	0.5785
GRU	0.6969	0.5788	0.6324
BiGRU	0.6968	0.6706	0.6835
CGRU	0.7260	0.7106	0.7182
Model	Precision	Recall	F1 Score

**Table 4:** CGRU model results for Z_135 and Z_15 datasets.

	Precision	Recall	F1 Score
Z_135	0.9361	0.9361	0.9361
Z_15	1	1	1
